# Cardiovascular benefits of GLP-1 receptor agonists in nonobese patients with HFpEF

**DOI:** 10.1097/MS9.0000000000004283

**Published:** 2025-11-11

**Authors:** Ehtisham Haider, Naveed Ahmad, Shamal Khan, Sakan Binte Imran

**Affiliations:** a Department of Medicine, Punjab Medical College, Faisalabad, Pakistan; b Department of Medicine, Khyber Medical College, Peshawar, Pakistan; c Department of Medicine, Sir Salimullah Medical College, Bangladesh

Heart failure with preserved ejection fraction (HFpEF) is a heterogeneous clinical problem that is age-related, systemic inflammatory, and metabolic, and is linked to high morbidity due to the lack of validated disease-modifying interventions^[1]^. In many randomized controlled trials (RCTs) and meta-analyses, GLP-1 receptor agonists (GLP −1 RAs) have been found to prevent major adverse cardiovascular events (MACE) and have positive effects on symptoms, functional capacity, and heart failure outcomes, most pronounced in obese or diabetic patients with HFpEF^[2]^. However, it remains unclear how much cardiovascular and symptomatic benefits of GLP-1 RAs can be seen in nonobese patients with HFpEF; subgroup and exploratory analyses have provided hints about heart failure hospitalization reduction, but these analyses are underpowered and highly confounded by weight loss and metabolic amelioration^[3]^. Therefore, special trials and mechanistic studies separating weight-independent cardiovascular processes, including anti-inflammatory effects, myocardial energetics, and endothelial actions are urgently needed. GLP-1 RA treatment will expand the existing HFpEF treatment approach to include more than symptom alleviation and allow metabolic-cardiac adjustment to be targeted across different body mass index groups^[4]^.

Evidence from large trials shows that GLP-1 RAs lower the risk of MACE, mainly in patients with obesity or type 2 diabetes, where metabolic effects such as weight reduction and improved insulin sensitivity play a major role^[2,3]^. However, nonobese patients with HFpEF lack this metabolic load, increasing uncertainty about whether they gain similar benefits^[5]^. Early subgroup analyses suggested slight reductions in heart failure hospitalization, regardless of baseline body mass index, although the findings were limited by small sample sizes and confounded by weight-related changes^[3,6]^. Beyond weight loss, GLP-1 RAs have systemic anti-inflammatory effects and enhance endothelial function, both highly relevant in HFpEF, where microvascular dysfunction and chronic inflammation contribute to progression^[1,4,6]^. Preclinical models also indicate improved myocardial efficiency through better substrate utilization, indicating potential benefits in nonobese patients independent of weight changes^[6]^. However, most RCTs, such as STEP-HFpEF, enrolled largely obese populations, limiting generalizability^[1]^. Observational analyses indicate possible additive effects when combined with SGLT2 inhibitors; however, data are still scarce for leaner cohorts^[5]^. Current meta-analyses suggest consistent cardiovascular protection in diabetes, regardless of baseline weight; however, trials designed for nonobese HFpEF remain absent^[2,3]^. This highlights a major evidence gap, where mechanistic studies are urgently needed to clarify weight-independent pathways^[4,6]^.

GLP-1 RAs have the potential to improve outcomes in HFpEF; however, current evidence largely reflects obese or diabetic cohorts. For nonobese patients, the benefits may extend beyond weight reduction and involve anti-inflammatory, vascular, and myocardial effects. However, the lack of targeted randomized trials limits this certainty. Future research must focus on weight-independent mechanisms, stratified HFpEF phenotypes, and combination therapies with SGLT2 inhibitors. Large, targeted trials in nonobese HFpEF patients are essential to establish whether GLP-1 RAs can modify the disease course in this high-risk group. This work has been written in accordance with the Titan guidelines (2025) ^[7]^.

## Data Availability

Data available on request from the authors.
